# Supplementary material for: Content-rich biological network constructed by mining PubMed abstracts
Source: BMC Bioinformatics. 2004 Oct 8;5:147. doi: 10.1186/1471-2105-5-147 (PMC528731; doi:10.1186/1471-2105-5-147)
Supplement: Additional File 2 — The original results of the above study (non-essential files are deleted to keep the file size under the limit set by BMC bioinformatics). [file 1471-2105-5-147-S2.bz2 › chilibotAdditionalFile2/dip50/400ID8955093E9955/html/LIF_LIFR.html]

 


 **LIF** and **LIFR** 
  
Found 183 abstracts in PubMed, retrieved 50.  
 

 What does Google say? 
 PDF only 
| .edu only 

---

**Interactive relationship** (e.g. stimulation, inhibition, etc)

**Inhibitory relationship**- The neurokine leukemia inhibitory factor  [ **LIF** ]   **LIF**  initiates signaling through heterodimerization of the low affinity  **LIF**  receptor  **LIFR**  and gp130.  Ref: 12425940 Brain Res Mol Brain Res, 2002
- The CDFs leukemia inhibitory factor  [ **LIF** ]  and ciliary neurotrophic factor bind to a  **LIFR**  gp130 receptor complex to activate Jak signal transducers and activators of transcription and Ras mitogen activated protein kinases signaling pathways.  Ref: 12609984 J Biol Chem< MedlineTA>J Biol Chem, 2002
- Similarly, smooth muscle cells in vitro treated with  **LIF**  exhibited a time dependent reduction in  **LIFR**  alpha protein expression and the subsequent reduction in transcription of the TIMP 1 gene.  Ref: 11795283 Ann N Y Acad Sci, 2001
- Leukemia inhibitory factor  [ **LIF** ]   **LIF**  promotes giant cell differentiation in vitro, and  **LIF**  receptor  **LIFR**  deficiency results in loss of giant cell differentiation in vivo.  Ref: 12554639 EMBO J, 2003
- The truncated forms of  **LIFR**  alpha subunit gp190EX and gp130 gp130EX which can compete with the wild type receptor for binding to leukemia inhibitory factor  [ **LIF** ]   **LIF**  were expressed on the membrane of U937 cells.  Ref: 11721361 Zhonghua Xue Ye Xue Za Zhi, 1999
**Neutral relationship**- Previous studies suggested that the NH 2 terminal CBM and the Ig like domain of the  **LIFR**  are most important for  **LIF**  binding and activity.  Ref: 12731865 Biochemistry, 2003
- Deletion mutants of the  **LIFR**  revealed that both the NH 2 terminal CBM and the Ig like domain of the  **LIFR**  are involved in  **LIF**  binding, presumably via the  **LIF**  site III epitope.  Ref: 11812136 J Mol Biol, 2002
- Continuous infusion of  **LIF**  30 microg kg day resulted in the downregulation of  **LIFR**  alpha in injured arteries in vivo.  Ref: 11795283 Ann N Y Acad Sci, 2001
- ... RILLIFdeltaII binds  **LIF**  and mediates signalling as efficiently as the wild type  **LIFR** .  Ref: 11812136 J Mol Biol, 2002
- Critical functional residues within the  **LIF**  sequence were also conserved including residues implicated in intracellular  **LIF**  activity, and in interaction with the receptor subunits  **LIFR**  and gp130.  Ref: 11435700 Cytogenet Cell Genet, 2001
- In analogy to a recently published model of the IL 6R complex, a model of the active  **LIFR**  complex is suggested which positions the COOH terminal CBM at  **LIF**  site I and the NH 2 terminal CBM and the Ig like domain at site III.  Ref: 11812136 J Mol Biol, 2002
- These data indicate that although  **LIF**  and Hyper CNTF use the same heterodimeric receptor complex of gp130 and  **LIFR** , only Hyper CNTF induces neuronal differentiation.  Ref: 12071967 Eur J Biochem, 2002
- Our results indicate that  **LIF** , CT 1 and OSM share an overlapping binding site located in the Ig like domain of  **LIFR** .  Ref: 12707269 J Biol Chem< MedlineTA>J Biol Chem, 2002
- Oncostatin M can activate two related receptors, one consisting of a low affinity  **LIF**  receptor subunit,  **LIFR**  beta, which forms a heterocomplex with the gp130 signal transducing protein and can recognize both oncostatin M and  **LIF** , and a second heterocomplex consisting of a subunit that specifically recognizes oncostatin M, OSMR beta, and the gp130 protein.  Ref: 12061840 Cytokine, 2002
- The different behavior of  **LIF**  and CT 1, on one side, and of OSM, on the other side, can be related to the different affinity of their site III for  **LIFR** .  Ref: 12707269 J Biol Chem< MedlineTA>J Biol Chem, 2002
- Signal transduction by CNTF requires that it bind first to CNTF R, permitting the recruitment of gp130 and  **LIF**  R  [ **LIFR** ] , forming a tripartite receptor complex.  Ref: 12765290 J Zhejiang Univ Sci< MedlineTA>J Zhejiang Univ Sci, 2002
- However, in the presence of an intact endothelium,  **LIFR**  alpha was upregulated in response to  **LIF** , and accordingly the downstream induction of iNOS expression was also increased.  Ref: 11795283 Ann N Y Acad Sci, 2001

**Non-interactive relationship** (e.g. studied together, co-existance, homology, etc.)

- While on BAF3 gp130  **LIF**  R  [ **LIFR** ]  cells, Hyper CNTF and  **LIF**  exhibited similar biological responses, the activity of Hyper CNTF on pheochromocytoma cells PC12 cells was quite distinct from that of  **LIF** .  Ref: 12071967 Eur J Biochem, 2002
- The mRNA levels of neuropoietic cytokines, ciliary neurotrophic factor CNTF, leukemia inhibitory factor  [ **LIF** ]   **LIF** , and interleukin 6 IL 6, and their receptor components CNTFR alpha,  **LIFR**  beta, IL 6R alpha, and gp130 were examined in seventy six patients with various peripheral neuropathies to determine the extent of expression of these cytokines and receptors, and their relationship to nerve fiber pathology and cell infiltration in the diseased nerves.  Ref: 11358282 Neurochem Res, 2001
- **LIF**  R  [ **LIFR** ]  and IP R activation induced binding of CREB and or ATF 1 to C EBP promoters and CREB dependent transcription.  Ref: 11682632 Mol Endocrinol, 2001
- gp130 either homodimerizes in response to IL 6 and IL 11 or forms heterodimers with the leukemia inhibitory factor  [ **LIF** ]   **LIF**  receptor  **LIFR**  in response to  **LIF** , oncostatin M OSM, ciliary neurotrophic factor CNTF, cardiotrophin 1 CT 1 or cardiotrophin like cytokine resulting in the onset of cytoplasmic tyrosine phosphorylation cascades.  Ref: 12047380 Eur J Biochem, 2002
- Pinopode formation and expression of  **LIF**  and  **LIFR**  were examined in endometrial samples.  Ref: 12620495 Fertil Steril, 2003
- Leukemia inhibitory factor  [ **LIF** ]  receptor  **LIFR** , epidermal growth factor receptor EGFR, and their respective ligands have been implicated in regulating growth and development of the early pig conceptus.  Ref: 12698007 Cytogenet Genome Res, 2002
- **LIF**  expression was up regulated transiently at 2 days post OBX, when up regulated expression of  **LIFR**  also was detected on globose basal cells GBCs, a subpopulation of which are immediate progenitors of olfactory receptor neurons.  Ref: 11370011 J Comp Neurol, 2001
- **LIF**  receptors, gp130 and  **LIFR**  alpha were significantly upregulated in cHI P &lt.  Ref: 12799551 AIDS< MedlineTA>AIDS, 2001
- Serum  **LIF**  R  [ **LIFR** ]  concentrations were significantly increased 2 and 4 months after starting treatment with atypical antipsychotics.  Ref: 11950553 Schizophr Res, 2002
- We measured plasma kynurenine and tryptophan and immune markers, such as serum interleukin 6 IL 6, IL 8 and the leukemia inhibitory factor  [ **LIF** ]  receptor  **LIF**  R  [ **LIFR** ]  in healthy, nonpregnant and pregnant women at the end of term and one and three days after delivery.  Ref: 12175700 Life Sci, 2002
- Normal human pituitary tissue expressed  **LIF** , as well as the  **LIF**  receptor  **LIFR**  signaling subunits, gp130 and  **LIFR** .  Ref: 12574225 J Clin Endocrinol Metab, 2003
- CONCLUSION S The simultaneous positive spatial and temporal expression of pinopodes and  **LIF**  and  **LIFR**  proteins in endometrial samples from healthy women suggests that both molecular and structural cell changes are important in the initiation of human blastocyst implantation.  Ref: 12620495 Fertil Steril, 2003
- Molecular modeling of the complex of  **LIF**  with the Ig like domain of  **LIFR**  provides a clue for the superadditivity of the D214A F284A double mutation.  Ref: 12707269 J Biol Chem< MedlineTA>J Biol Chem, 2003
- In addition, the expression of ciliary neurotrophic factor receptor CNTFR and leukemia inhibitory factor  [ **LIF** ]  receptor  **LIFR**  mRNAs, but not of interleukin 6 receptor mRNA, was up regulated after systemic lipopolysaccharide treatment.  Ref: 12127095 Neuroscience, 2002
- On the same days, blood samples were collected for each patient to measure serum concentrations of cytokines IL 6, IL 10, IL 1ra and cytokine receptors sIL 2R,  **LIF**  R  [ **LIFR** ] .  Ref: 11585680 Psychoneuroendocrinology, 2001
- Cells that only express gp130 and  **LIF**  R  [ **LIFR** ] , but not CNTF R are refractory to stimulation by CNTF.  Ref: 12765290 J Zhejiang Univ Sci< MedlineTA>J Zhejiang Univ Sci, 2001
- The functional receptor complex of CNTF is composed of the CNTF receptor alpha CNTFR, gp130 and the leukemia inhibitory factor  [ **LIF** ]  receptor  **LIFR** .  Ref: 11943154 FEBS Lett, 2002
- We concluded that the fusion protein Hyper CNTF had superagonistic activity on target cells expressing gp130 and  **LIF**  R  [ **LIFR** ] , but lacking membrane bound CNTF R.  Ref: 12765290 J Zhejiang Univ Sci< MedlineTA>J Zhejiang Univ Sci, 2002
- Human CNTF elicits cellular responses by induction of a receptor complex consisting of the CNTF alpha receptor CNTFR, which is not involved in signal transduction, and the beta receptors gp130 and leukemia inhibitory factor  [ **LIF** ]  receptor  **LIFR** .  Ref: 12643274 J Biol Chem, 2003
- This study focuses on the expression of the neuropoietic cytokines leukemia inhibitory factor  [ **LIF** ]   **LIF**  and its receptor  **LIFR**  and interleukin 6 IL 6 and its receptor IL 6R in intercellular signaling pathways in the olfactory mucosa after target ablation.  Ref: 11370011 J Comp Neurol, 2001
- This study was carried out to examine 1 the serum concentrations of interleukin 8 IL 8, IL 10, the soluble CD8 sCD8 and the leukemia inhibitory factor  [ **LIF** ]  receptor  **LIF**  R  [ **LIFR** ]  in nonresponders to treatment with typical neuroleptics as compared with normal volunteers and responders to treatment.  Ref: 11950553 Schizophr Res, 2002
- Mutation of a specific cysteine in D5 of gp130 C458A leads to constitutive heterodimerization with the  **LIFR**  and increased sensitivity towards  **LIF**  stimulation.  Ref: 12047380 Eur J Biochem, 2002
- These results show that even though human prolactinomas express gp130 and  **LIFR** , and respond to exogenous  **LIF** , albeit less than normal lactotrophs, they are largely devoid of  **LIF** .  Ref: 12574225 J Clin Endocrinol Metab, 2003
- For signal transduction, CNTF requires a receptor complex, composed of the IL 6 signal transducing molecule gp130, leukemia inhibitory factor  [ **LIF** ]  receptor  **LIFR**  beta, and CNTFR alpha.  Ref: 11464862 Med Pediatr Oncol, 2001
- Signal of the cytoplasmic regions of leukemia inhibitory factor  [ **LIF** ]  receptor  **LIFR**  alpha subunit and gp130 involves Stat3 activation in leukemic U937 cells < ArticleTitle>.  Ref: 11721361 Zhonghua Xue Ye Xue Za Zhi, 1999
- The mRNAs for  **LIF**   **LIFR** , IL 6 IL 6R, and their common signal transduction molecule, gp130, in olfactory nasal mucosa from control mice and from 3 day post OBX mice were detected with reverse transcriptase polymerase chain reaction RT PCR.  Ref: 11370011 J Comp Neurol, 2001
- The proliferative results confirmed that CNTF required homodimerization of the gp130, CNTF R and  **LIF**  R  [ **LIFR** ]  receptor subunit whereas Hyper CNTF required heterodimerization of the gp130 and  **LIF**  R  [ **LIFR** ]  receptor subunit.  Ref: 12765290 J Zhejiang Univ Sci< MedlineTA>J Zhejiang Univ Sci, 2001
- The present study demonstrates an upregulation of both the  **LIF**  receptor  **LIFR**  alpha subunit and the signal transducing subunit gp130 following endothelial denudation of the carotid artery by balloon catheter.  Ref: 11795283 Ann N Y Acad Sci, 2001
- To elucidate the function of the two cytokine binding modules CBM of the leukemia inhibitory factor  [ **LIF** ]  receptor  **LIFR** , receptor chimeras of  **LIFR**  and the interleukin 6 receptor IL 6R were constructed.  Ref: 11812136 J Mol Biol, 2002
- Although all of 19 prolactinomas tested expressed gp130 and  **LIFR**  subunit mRNA and immunoreactive protein, only 3 of 19 prolactinomas expressed  **LIF**  mRNA.  Ref: 12574225 J Clin Endocrinol Metab, 2003
- Here we report that the expression of leukemia inhibitory factor  [ **LIF** ]  receptor  **LIFR** , one of the signaling molecules shared by several neuropoietic cytokines of the IL 6 family, is specifically upregulated in PC12 cells following treatment with NGF.  Ref: 12871977 J Biol ChemJ Biol Chem, 2003
- and iii the relationships between the availability of plasma tryptophan to the brain and inflammatory markers, such as serum interleukin 6 IL 6, interleukin 1 receptor antagonist IL 1RA and the leukaemia inhibitory factor receptor  **LIF**  R  [ **LIFR** ] .  Ref: 11459382 Psychol Med, 2001
- Either the NH 2 terminal chimera RILLIFdeltaI or the COOH terminal  **LIFR**  CBM chimera RILLIFdeltaII were replaced by the structurally related CBM of the IL 6R which does not bind  **LIF** .  Ref: 11812136 J Mol Biol, 2002
- Expression of mRNAs for ciliary neurotrophic factor CNTF, leukemia inhibitory factor  [ **LIF** ]   **LIF** , interleukin 6 IL 6, and their receptors CNTFR alpha,  **LIFR**  beta, IL 6R alpha, and gp130 in human peripheral neuropathies.  Ref: 11358282 Neurochem Res, 2001
- The functional receptor complex of ciliary neurotrophic factor CNTF, a member of the gp130 family of cytokines, is composed of CNTF, the CNTF receptor alpha CNTFR, gp130 and the leukemia inhibitory factor  [ **LIF** ]  receptor  **LIFR** .  Ref: 12707266 J Biol Chem< MedlineTA>J Biol Chem, 2001
- Continuous treatment with Dex 10 8 mol L inhibited the endogenous production of IL 6,  **LIF** , OSM, IL 11R, and OSMR, but had no detectable effect on the expression of IL 11, CT 1, CNTF, CNTFR,  **LIFR** , or gp130.  Ref: 12110437 Bone, 2002
- The main function of the COOH terminal CBM of the  **LIFR**  is to position the NH 2 terminal CBM and the Ig like domain, so that these can bind to  **LIF** .  Ref: 11812136 J Mol Biol, 2002
- Flow cytometric analyses detected both the OSM specific receptor and the gp130 receptor at the chondrocyte cell surface, but failed to detect the leukemia inhibitory factor  [ **LIF** ]  receptor  **LIFR** .  Ref: 11465713 Arthritis Rheum, 2001
- Both surface and glandular epithelial cells expressed maximal levels of  **LIF**  and  **LIFR**  protein, in biopsy samples showed fully developed pinopodes.  Ref: 12620495 Fertil Steril, 2003
- Here we studied the expression of cytokines IL 6,  **LIF** , IL 1beta and TNFalpha and cytokine receptors IL 6R,  **LIFR**  and Gp130 in the rat brain after kainic acid induced status epilepticus using Northern blot analysis and in situ hybridization histochemistry.  Ref: 12591161 Brain Res Mol Brain Res, 2003
- In preadipocytes, their expression is up regulated by the activation of leukemia inhibitory factor  [ **LIF** ]  receptor  **LIF**  R  [ **LIFR** ]  and prostacyclin receptor IP R via the extracellular signal regulated kinase ERK pathway and cAMP production, respectively.  Ref: 11682632 Mol Endocrinol, 2001
- The leukemia inhibitory factor  [ **LIF** ]   **LIF** , cardiotrophin 1 CT 1 and oncostatin M OSM are four helix bundle cytokines acting through a common heterodimeric receptor composed of gp130 and  **LIF**  receptor  **LIFR** .  Ref: 12707269 J Biol Chem< MedlineTA>J Biol Chem, 2001
- Mapping and microsatellite marker development for the porcine leukemia inhibitory factor  [ **LIF** ]  receptor  **LIFR**  and epidermal growth factor receptor EGFR genes.  Ref: 12698007 Cytogenet Genome Res, 2002
- Before and after the appearance of pinopodes,  **LIF**  and  **LIFR**  immunostaining was less intense or faint.  Ref: 12620495 Fertil Steril, 2003
- **LIF**  was up regulated in the hippocampus, cortex and meninges after seizures, and  **LIFR**  mRNA was induced in the hippocampus and cortex.  Ref: 12591161 Brain Res Mol Brain Res, 2003
- The heterodimeric cytokine composed of the soluble ciliary neurotrophic factor receptor sCNTFR and the IL 6 family member cardiotrophin like cytokine CLC was recently identified as a new ligand for gp130 leukemia inhibitory factor  [ **LIF** ]  receptor  **LIFR**  complex Plun Favreau, H.  Ref: 11952795 Eur J Biochem, 2002
- Increased proliferation of parietal endoderm in  **lifr**  embryoid bodies and addition of conditioned  **lif**  cell culture supernatant promoted cardiomyogenesis, demonstrating for the first time that parietal endoderm also contributes to cardiomyogenesis in embryoid bodies in a paracrine and leukemia inhibitory factor  [ **LIF** ]  and its receptor independent pathway.  Ref: 11683491 Differentiation, 2001
- OBJECTIVE To determine cell type specific expression of leukemia inhibitory factor  [ **LIF** ]   **LIF**  and  **LIF**  receptor  **LIFR**  proteins relative to formation of pinopodes in human endometrial samples.  Ref: 12620495 Fertil Steril, 2003
- Hyper CNTF efficiently induced dose dependent STAT3 phosphorylation and proliferation of BAF 3 cells stably transfected with gp130 and  **LIF**  R  [ **LIFR** ]  cDNAs.  Ref: 12071967 Eur J Biochem, 2002
- Because corticosterone is known to have a broad range of effects and  **LIF**  function has been associated with the hypothalamo pituitary adrenal axis, this study was designed to determine the role for  **LIFR**  in the fetus when exposed to the elevated maternal glucocorticoid levels of late gestation.  Ref: 12823859 Reprod Biol Endocrinol< MedlineTA>Reprod Biol Endocrinol, 2002
- Here, we report the construction and expression of a CNTF soluble CNTF receptor sCNTF R fusion protein Hyper CNTF with enhanced biological activity on cells expressing gp130 and leukemia inhibitory factor  [ **LIF** ]  receptor  **LIF**  R  [ **LIFR** ] , but not membrane bound CNTF R.  Ref: 12071967 Eur J Biochem, 2002
- 2 prolonged treatment with atypical antipsychotics may increase the anti inflammatory capacity of the serum in schizophrenic patients by increasing serum  **LIF**  R  [ **LIFR** ]  concentrations.  Ref: 11950553 Schizophr Res, 2002
- Relative quantitative RT PCR was used to demonstrate that within hours of OBX, mRNAs encoding three key components in the leukemia inhibitory factor  [ **LIF** ]   **LIF**  signaling pathway, including  **LIF** ,  **LIF**  receptor  **LIFR** , and STAT3, as well as cyclin D1, a growth factor sensor indicative of progenitor cell transformation, were upregulated.  Ref: 11782968 J Neurosci Res, 2002
- Using RT PCR we detected transcripts of the receptor components involved in OSM signaling, gp130, OSM receptor OSMR beta, and leukemia inhibitory factor  [ **LIF** ]  receptor  **LIFR** , in HCEC.  Ref: 11706938 J Neuropathol Exp Neurol, 2001
- BACKGROUND Ablation of the low affinity receptor subunit for leukemia inhibitory factor  [ **LIF** ]   **LIFR**  causes multi systemic defects in the late gestation fetus.  Ref: 12823859 Reprod Biol Endocrinol< MedlineTA>Reprod Biol Endocrinol, 2001
- In contrast to other hematopoietic cytokine receptors, the leukemia inhibitory factor  [ **LIF** ]  receptor  **LIFR**  possesses two cytokine binding modules CBMs.  Ref: 12731865 Biochemistry, 2003
- In contrast, the mRNA levels of  **LIF** , IL 6 and the ligand binding receptor components CNTFR alpha,  **LIFR**  beta and IL 6R alpha were elevated to variable extent in the diseased nerves.  Ref: 11358282 Neurochem Res, 2001
- BACKGROUNDLeukemia inhibitor factor  **LIF** , a member of the interleukin 6 cytokine family, has recently been shown to inhibit HIV 1 replication both in vivo and in vitro.OBJECTIVELIF and its corresponding receptors gp130 and  **LIF**  receptor alpha  **LIFR**  alpha were studied in lymphoid tissue LT to reveal potential systemic immunoregulatory effects during the course of HIV 1 infection.METHODSLIF, gp130,  **LIFR**  alpha and HIV 1 replicating cells were identified at the single cell level by immunohistochemistry and quantified by computerized in situ imaging in tonsil and lymph nodes biopsies LT from patients with primary HIV 1 infection PHI, chronic HIV 1 infection cHI, long term non progressors LTNP and HIV 1 seronegative controls.RESULTSLIF and its receptors, gp130 and  **LIFR**  alpha were significantly P &lt.  Ref: 12799551 AIDS< MedlineTA>AIDS, 2001
- Finally, treatment with exogenously added  **LIF**  stimulated IL 6,  **LIF** ,  **LIFR** , and OSMR, but had no other detectable effects.  Ref: 12110437 Bone, 2002
- The aim of this study was to assess the expression and regulation of  **LIF**  receptor  **LIFR**  and gp130 mRNA in mouse uterus during early pregnancy using in situ hybridization and reverse transcription polymerase chain reaction RT PCR.  Ref: 12203823 Mol Reprod Dev, 2002
- In the olfactory nerve ON,  **LIF**  positive and IL 6 positive macrophage infiltration was followed by the transient up regulation of expression of  **LIFR** , IL 6, and IL 6R in ensheathing cells by 3 days post OBX.  Ref: 11370011 J Comp Neurol, 2001
- The changes in the tryptophan CAA ratio from the end of term to the early puerperium were significantly and inversely related to serum IL 6, IL IRA and  **LIF**  R  [ **LIFR** ] .  Ref: 11459382 Psychol Med, 2001
